# A multi-centre, pragmatic, three-arm, individually randomised, non-inferiority, open trial to compare immediate orally administered, immediate topically administered or delayed orally administered antibiotics for acute otitis media with discharge in children: The Runny Ear Study (REST): study protocol

**DOI:** 10.1186/s13063-020-04419-7

**Published:** 2020-06-03

**Authors:** Kathryn Curtis, Michael Moore, Christie Cabral, Vasa Curcin, Jeremey Horwood, Richard Morris, Vibhore Prasad, Anne Schilder, Nicholas Turner, Scott Wilkes, Alastair D. Hay, Jodi Taylor

**Affiliations:** 1 Centre for Academic Primary Care, Population Health Sciences, Bristol Medical School, University of Bristol, Canynge Hall, 39 Whatley Road, Bristol, BS82PS UK; 2 Primary Care, Population Sciences and Medical Education, Faculty of Medicine, University Of Southampton, Southampton, SO17 1BJ UK; 3 School of Population Health and Environmental Sciences, Faculty of Life Sciences and Medicine, King’s College London, Addison House 3.07, Guy’s Campus, London, SE1 1UL UK; 4 evidENT, UCL Ear Institute, Royal National Throat, Nose and Ear Hospital, 330 Grays Inn Road, London, WC1X 8DA UK; 5 Bristol Randomised Trial Collaboration (BRTC), part of the Bristol Trial Centre, Bristol Medical School, University of Bristol, Canynge Hall, 39 Whatley Road, Bristol, BS82PS UK; 6 School of Medicine, Faculty of Health Sciences and Wellbeing, University of Sunderland, Sciences Complex, City Campus, Chester Road, Sunderland, SR1 3SD UK

**Keywords:** Acute otitis media, Primary care, Antibiotics, Paediatrics, Randomised controlled trial

## Abstract

**Background:**

Acute otitis media (AOM) is a common painful infection in children, with around 2.8 million cases presenting to primary care in England and Wales annually. Nearly all children who present to their general practitioner (GP) with AOM or AOM with discharge (AOMd) are treated with orally administered antibiotics. These can cause side effects; contribute to the growing problem of antimicrobial resistance, and more rarely, allergic reactions. Alternative treatments, such as an antibiotic eardrops, or ‘delayed’ orally administered antibiotics, could be at least as effective and safe as immediate orally administered antibiotics for children with AOMd.

**Methods/design:**

REST is a pragmatic, three-arm, individually randomised, non-inferiority trial being conducted in 175 GP practices across the United Kingdom (UK). The study aims to recruit 399 children aged (≥ 12 months and < 16 years) presenting to their GP with AOMd. Children will be randomised to one of three arms: immediate ciprofloxacin 0.3% eardrops; delayed orally administered amoxicillin (clarithromycin if penicillin allergic) or immediate orally administered amoxicillin (clarithromycin).

Recruitment, including eligibility screening, randomisation and data collection, are conducted using the innovative, TRANSFoRm electronic trial management platform. Integrated within the primary care electronic medical records it provides automatic eligibility checking, part-filling of e-CRFs, study workflow management and routine NHS follow-up data collection. The primary outcome is time to resolution of all significant symptoms and will be collected by the parent using a Symptom Recovery Questionnaire (SRQ). Secondary outcomes, including cost-effectiveness, duration of moderately bad or worse symptoms and repeat AOMd episodes, will be collected at day-14 and at 3 months.

**Discussion:**

It is unclear whether prescribing orally administered antibiotics to children with AOMd results in a reduction in symptoms or a shorter duration of illness. The REST trial should allow us to compare the non-inferiority of: immediate topically administered ciprofloxacin ear drops, or delayed orally administered amoxicillin (clarithromycin) against immediate orally administered amoxicillin (clarithromycin). We aim to recruit 399 patients from 175 practices in the UK. Using the TRANSFoRm software to randomise participants to the trial will enable recruitment for a relatively uncommon condition.

**Trial registration:**

Name of Registry: ISCRTN

Registration Number: ISRCTN12873692. This contains all items required to comply with the World Health Organization Trial Registration Data Set

Date of Registration: 24 April 2018

Name of Registry: EudraCT

Registration Number: 2017-003635-10

Date of Registration: 6 September 2017

## Background and rationale

Acute otitis media (AOM) is important to children, parents and the National Health Service (NHS). Firstly, the infection causes pain and distress to the child, disrupting sleep and family routines. In around 15% of cases, a rise in middle-ear pressure and/or inflammation weakening the tympanic membrane results in it bursting, discharging pus from the middle ear as a discharge (otorrhoea) [1]. Children with AOM and discharge (AOMd) have similar levels of pain and are more unwell at presentation than children with AOM [2, 3]. Moreover, children with AOMd have a worse prognosis, and higher rates of pain at 1 week, as reported by parents (carers), repeat AOM episodes and hearing problems at 3 months [2]. Estimates of parental costs (travel, over the counter (OTC) medicines and lost earnings) vary [4–6], with even the lowest suggesting £4 million in England and Wales per annum.

Over 90% of UK parents attend primary care health services for each episode of AOMd [7], with over 150,000 general practitioner (GP) consultations for AOMd in England and Wales per annum at a cost to the NHS of over £3 million [4, 5], which is more than for any other common symptom of acute infection.

More children with AOM and AOMd receive an orally administered antibiotic in the UK [8] and the United States [9] than for any other respiratory infection, with three quarters of GPs prescribing orally administered antibiotics to at least 80% of children diagnosed as such [10, 11]. Our 2015 audit, including 33 GP practices and 56,251 children, confirmed that immediate orally administered antibiotics is usual care for AOMd: 88% were given orally administered antibiotics of which 97% were given immediately.

There is strong evidence that children with AOMd benefit from immediate orally administered antibiotics. The number needed to treat with antibiotics is three to reduce the proportion of children with pain and/or fever at 3–7 days compared with placebo/no treatment [3]. The National Institute for Health and Care Excellence (NICE), therefore, recommends that immediate antibiotics should be considered [12]. Orally administered antibiotics do, however, also cause side effects, are associated with subsequent eczema and hay fever [13] and with population [14] and patient-level [15] antimicrobial resistance.

In response to the UK’s Antimicrobial Resistance Action Plan calls for research to preserve antibiotic effects [16] we have designed a trial of topically administered antibiotics in children with AOMd, aiming to reduce the use of systemic antibiotics. Perforation of the tympanic membrane provides a portal of entry into the middle ear for antibiotic drops instilled in the ear canal. In children with ventilation tubes (‘grommets’), it has been shown that topically administered antibiotics can reach the infected middle ear despite purulent discharge [17], and that compared with orally administered antibiotics, they are more effective in reducing the duration of otorrhoea, recurrence of AOM and have fewer side effects [17]. This study also showed that topically administered antibiotics are cost-effective [18]. However, further research is needed in children with AOMd without grommets, since the opening to the middle ear may be smaller and the tympanic membrane heals quickly, which could prevent the drops from reaching the middle ear. If topically administered and delayed antibiotics are shown to be non-inferior, we also need to understand the acceptability of such treatment to clinicians and parents and how to address any barriers to implementation.

We will address this evidence gap by assessing the clinical effectiveness and economic implications of immediate topically vs. delayed orally administered antibiotics and testing the hypotheses: (1) immediate antibiotics are better than placebo/no treatment for AOMd symptoms [3] and (2) delayed orally administered antibiotics are similar to immediate orally administered antibiotics in children with AOM (though with reduced antibiotic consumption) [1].

The REST study is a three-arm, randomised controlled trial (RCT) investigating the clinical effectiveness and economic implications of topically administered or delayed antibiotics compared with immediate orally administered antibiotics, powered for the duration and severity of the symptoms most important to parents, while also investigating adverse events, complications and AOM/AOMd recurrence. By testing two interventions that could reduce systemic antibiotic exposure (immediate topically administered and delayed orally administered antibiotics), this study is at the forefront of research to improve antimicrobial stewardship in AOMd.

## Methods/design

### Aims and objectives

The key aim of this research is to investigate the clinical effectiveness and economic impact of immediate topically or delayed orally administered antibiotics compared with immediate orally administered antibiotics for symptom duration in children presenting to primary care with AOM with discharge (AOMd).

Secondary objectives are to:

- Estimate the short-term cost-implications of immediate topically or delayed orally administered antibiotics compared with immediate orally administered antibiotics from the perspective of the NHS
- Compare the effects on duration of ‘moderately bad or worse’ symptoms; parent/legal guardian satisfaction with treatment; and adverse events
- Compare hearing loss and rate of recurrence of AOM/AOMd at 3 months
- Understand parent/legal guardian and clinician views of participating in a trial about AOMd, adherence and satisfaction with allocated treatment.
- Evaluate the impact of treatment on carriage of antibiotic resistance in the gut

### Trial design

This is a multi-site, pragmatic, three-arm, individually randomised (stratified by age < 2 vs. ≥ 2 years), non-inferiority, open trial. We compare (1) immediate topically administered ciprofloxacin 0.3% drops with (2) delayed orally administered antibiotics or (3) immediate orally administered antibiotic in children aged 12 months to 16 years with unilateral AOMd as the presenting symptom of recent-onset (≤ 7 days) AOM. The primary endpoint is collected by questionnaire at day 14 post recruitment. Secondary outcomes are collected both at 14 days and at 3 months by questionnaire.

This study is classified as a Type A study (low risk) by the Medicines and Healthcare Regulatory Agency (MHRA), with regulatory approval obtained on 4 May 2018.

The trial design includes an internal pilot recruitment phase of 6 months’ duration, primarily to verify that recruitment was possible before progression to the main phase of the trial.

The REST study utilises an integrated electronic trial management platform, TRANSFoRm, that was initially developed as part of the EU FP7 TRANSFoRm project (2009–2015) and evaluated in a 60-site clinical trial in Poland [19]. The system integrates as a plug-in within the host Electronic Health Record (EHR) system through the provider’s Application Programmer’s Interface (API). The key features of the system include:

- Automated eligibility checking; the TRANSFoRm plug-in allows the EHR opened during a consultation to be automatically checked against the REST eligibility criteria
- Consent – the TRANSFoRm platform allows the clinician to print the study consent form for the participant to sign a record of consent that can then be entered onto the platform initiating the trial’s workflow
- An integrated randomisation system for immediate randomisation of participants during consultation
- Trial-specific Electronic Case Report Forms (eCRFs) that are presented to clinicians at the appropriate appointments to complete. Some trial data is automatically retrieved from the SystmOne health record and used to part-fill the trial eCRF, which can be amended by the user

**Fig. 1:**
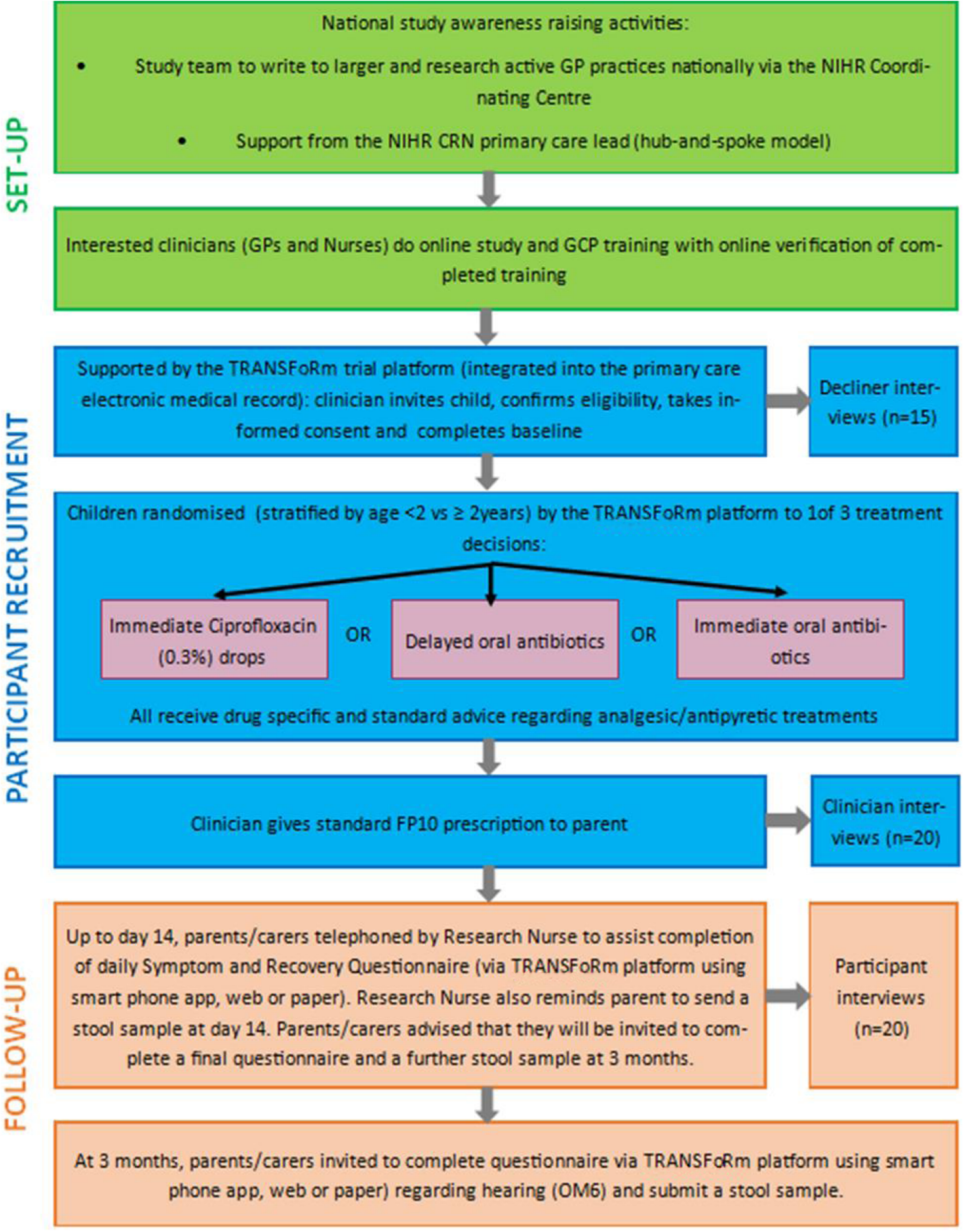
Trial flow chart

Upon submission of the eCRF the TRANSFoRm platform automatically records a record of trial activity in the participant’s health record. The study flow diagram is provided in Fig. 1 and the Standard Protocol Items: Recommendations for Interventional Trials (SPIRIT) Checklist in Additional file 1.

### Setting

We aim to recruit 175 SystmOne GP practices from all 15 UK CRN’s. The Clinical Research Network (CRN) invite research active SystmOne GP practices to express an interest in taking part in the study.

### GP practice set-up

Interested practices are sent a local document pack by the study team and practices are asked to return a set of documents including a CV, Good Clinical Practice (GCP) certificates and a signed site contract. Sites undergo remote training in trial conduct and the use of the TRANSFoRm electronic platform.

A site is only greenlighted once all local documents have been completed and the principal investigator (PI) has completed the online training. At this point the TRANSFoRm electronic platform is installed onto the PI’s computer. Once a site is greenlighted, additional clinicians from that practice can complete the REST training package, be added to the site delegation log and the TRANSFoRm platform can be downloaded onto additional practice computers.

### Participants and eligibility

Eligible participants are children aged ≥ 12 months to < 16 years whose parents/legal guardians are seeking primary medical care for acute-onset unilateral otorrhoea as the presenting symptom of recent-onset (≤ 7 days) AOM. Detailed inclusion and exclusion criteria are detailed in Table 1.

**Table 1:** Inclusion and exclusion criteria

Patient selection criteria	
**Inclusion criteria (child must meet all criteria):**	1. Children aged ≥ 12 months to < 16 years 2. Presenting with recent-onset (≤ 7 days) unilateral AOM with recent-onset (≤ 7 days) otorrhoea currently visible (or seen by parent/legal guardian ≤ 24 h) 3. Child attending with parent/legal guardian who is legally able to give consent in person 4. Parent/legal guardian willing and able to administer eardrops 5. Parent/legal guardian willing, able and available to complete the daily SRQ and received regular telephone calls from the study team
**Exclusion criteria (excluded if child meets any criterion at the time of entry):**	1. Symptoms/signs suggestive of bilateral AOM/AOMd 2. Child has symptoms/signs suggestive of serious illness and/or complications, e.g. mastoiditis and/or requires immediate hospitalisation 3. Child requires immediate orally administered antibiotics (e.g. for another infection or AOMd considered severe) 4. As per NICE guidelines [12], a child at high risk of serious complications: • Significant immunosuppression • Heart, lung, renal, liver or neuromuscular disease (defined as requiring ongoing inpatient or outpatient care from specialist teams) co-morbidities • Trisomy 21 (Down’s syndrome), cystic fibrosis or craniofacial malformation, such as cleft palate (these children are known to be at higher risk of AOM) 5. Grommet (ventilation tube) in situ in the ear with otorrhoea 6. Currently taking orally (for a respiratory tract infection) or topically administered (in the affected ear) antibiotics 7. Allergy to ciprofloxacin 8. Allergy to penicillin/anaphylaxis to another beta-lactam agent and allergy to clarithromycin 9. Child has taken part in any research involving medicines within the last 90 days 10. Child has already participated in this trial

*AOM* acute otitis media, *SRQ* Standard Recovery Questionnaire

### Patient screening and recruitment

The process of patient screening and recruitment is detailed below:

1. Children aged ≥ 12 months to < 16 years accompanied by a parent/legal guardian present to their GP with suspected AOMd
2. GP invites parent and child to participate in the REST study and provides a parent information leaflet (child information leaflet for children over 6 years old)
3. GP seeks verbal agreement from parents and assesses the child for eligibility using the TRANSFoRm electronic platform
4. Informed consent/assent is sought from parents of eligible children, baseline data and contact details are collected via the TRANSFoRm platform
5. Child is randomised using the TRANSFoRm platform and, if allocated, the drops or immediate antibiotics on an FP10 prescription (Standard UK prescription) are issued.

### Randomisation

After confirming eligibility and obtaining informed consent, participants will be randomised (stratified by age) to either (1) immediate topically administered ciprofloxacin 0.3% drops with (2) delayed orally administered antibiotics or (3) immediate orally administered antibiotics.

The randomisation sequence is generated by the Bristol Randomised Trials Collaboration (BRTC) and supplied to the TRANSFoRm electronic platform to be allocated to each successive participant recruited. A system for checking the correct randomisation allocation is built in to the TRANSFoRm platform. Clinicians will not be able to determine treatment allocation pre-randomisation.

### Consent

Parent Information Sheets (Additional file 2) will be given to the parents of potentially eligible children and discussed before consent is sought. Informed consent will be obtained from the parent or legal guardian of each child. Assent will be obtained from all children over the age of 6 years. Parents will also be given be the opportunity to consent to stool-sample collections (with the additional option of the sample being retained for future research in microbial infections), declining to consent to this element of the study will not exclude participation from other elements of the study. Parents will also be given the opportunity to consent to information being collected about their child being used to support other research in the future, and to this being shared anonymously with other researchers.

### Withdrawal

Participants remain in the trial unless they choose to withdraw, or if they are unable to continue. Parents can choose to completely withdraw their child or to withdraw from specific elements of the study without giving a reason. Any data collected up until this point will be retained for analysis. Information regarding the withdrawal criteria is detailed in the parent information leaflet.

### Interventions

#### Choice of comparator

We selected ciprofloxacin 0.3% drops as our topically administered antibiotic because it:

- Is active against all common otopathogens [2]
- Is non-ototoxic
- Is widely and routinely available in the UK
- Is colourless so will not interfere with assessing otorrhoea
- Will provide complementary evidence to the ZonMw-funded trial, which is using an antibiotic-steroid combination

We decided to avoid aminoglycoside drops because of concerns about potential ototoxicity. We have proposed delayed orally administered antibiotics as the second intervention since our previous trials [1, 20, 21] have achieved significant reductions in orally administered antibiotic consumption compared with immediate antibiotic prescribing (and similar symptom relief). Immediate orally administered amoxicillin (clarithromycin if allergic to penicillin) is the comparator as it reflects usual care and is well-tolerated.

#### Intervention description

Arm 1: (control) current usual care – orally administered amoxicillin suspension three times daily for 7 days (or orally administered clarithromycin twice daily for 7 days if allergic to penicillin).

Arm 2: (intervention) antibiotic drops, to be instilled three times daily into the discharging ear. Parents will be given written advice regarding how to administer the drops. This will include: (1) cleaning the outer ear of discharge that can be easily removed with a tissue; (2) tilting the child’s head to one side (to approximately 90°) when applying the eardrops; and (3) maintaining the tilt for a few minutes to improve penetration of the drops.

Arm 3:(intervention) a ‘delayed’ prescription for orally administered amoxicillin suspension antibiotics three times daily for 7 days (or orally administered clarithromycin twice daily for 7 days if allergic to penicillin). Parents will be given written advice to delay the prescription will consist of: (1) advising that the prescription is only ‘dispensed’ at a pharmacy if symptoms worsen or are not starting to improve by 4 days; and (2) safety-netting advice regarding the symptoms that should prompt review consultation (increasing pain, high temperatures, headaches, irritability or reduced feeding).

All groups will also receive standard advice to complete the antibiotic course and how to manage pain, fever and other symptoms (e.g. use of paracetamol/ibuprofen).

#### Post-trial care

Following participation in the study, children are returned to usual care by their GP. All participants will receive a summary of the results of the trial.

### Outcome measures

The primary outcome measure is time to resolution of the following symptoms: pain, fever, being unwell, sleep disturbance, otorrhoea and episodes of distress. The primary outcome is the time until all symptoms are rated by parents as ‘no’ or ‘very slight’ problem. This will be recorded by parents in the Symptom Recovery Questionnaire (SRQ).

Secondary outcome measures:

- Duration of ‘moderately bad or worse’ symptoms (pain, fever, being unwell, sleep disturbance, otorrhoea; episodes of distress/crying
- Appetite and interference with normal activities up to 14 days
- Antibiotic and analgesic use
- Adverse events – diarrhoea, rash, vomiting, serious complications
- Treatment adherence
- Parent/legal guardian satisfaction with treatment
- NHS resource use at 14 days
- Repeat AOM and AOMd episodes, serious complications and the OM6 hearing questionnaire at 3 months;
- Qualitative evaluation of recruitment, medication satisfaction, adherence and follow-up
- Analysis of stool sample to assess burden of resistance

### Assessment and follow-up

**Fig. 2:**
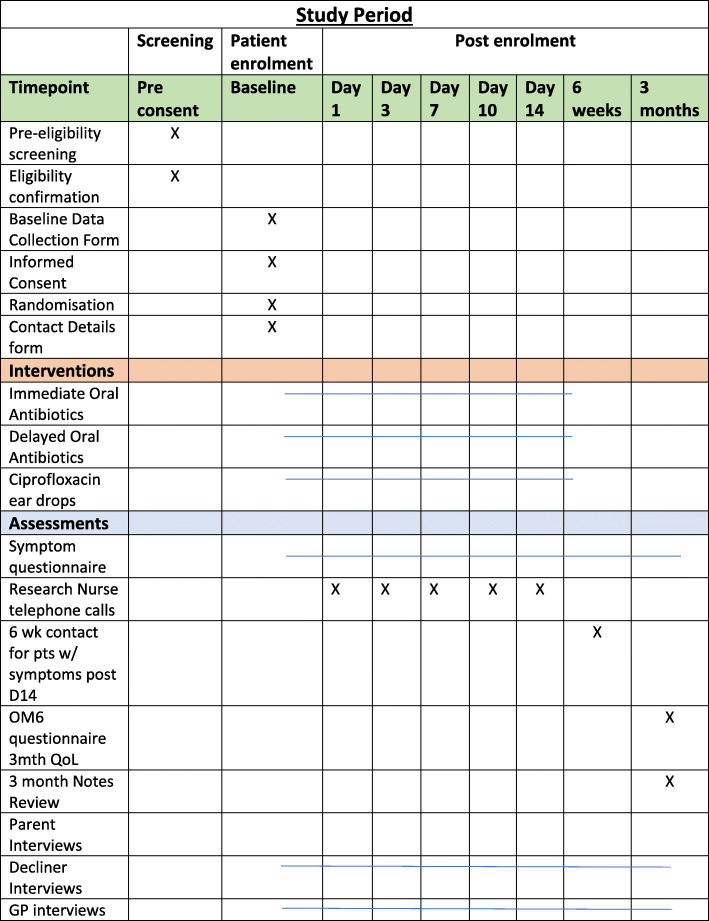
Participant recruitment and follow-up timeline. *Completed daily from day 1 to day 14

The components and timing of follow-up measures are shown in Fig. 2.

#### Clinician-recorded outcomes: baseline data collection form and contact form

During the consultation, clinicians will complete a baseline data collection form and contact detail form for all eligible participants. The baseline data collected includes acute clinical data and any relevant medical history relating to AOMd incidence.

A contact details form will record information from the parent/legal guardian including name, address, telephone number and availability to take calls from the research nurse.

#### Patient-reported outcomes

All parents/legal guardians are asked to complete a daily SRQ recording the symptoms identified by parents/legal guardians as important. The SRQ will be provided in electronic format via the TRANSFoRm app or in a paper version. The SRQ will provide a daily record of symptom burden and will be completed up to 14 days. The primary outcome will be collected using the SRQ with research nurse telephone calls on days 1, 3, 7, 10 and 14.

On day-7 and day-14 telephone calls, information on the use of healthcare resources including information about primary care contacts, use of 111, walk-in centres and hospital services will be obtained.

At month 3, use of hospital services will be collected by review of the patients’ EHR.

The final questionnaire will be sent 3 months after randomisation either electronically (web or iOS/Android app) via the TRANSFoRm platform or paper questionnaire. The questionnaire will ask about parent/legal guardian-reported hearing loss at 3 months as measured using the OM6 [22] questionnaire.

#### Storage and analysis of microbiological clinical samples

Plans for the collection, storage and evaluation of biological specimens can be found in Additional file 3.

### Economic data collection

The economic evaluation will explore the relationship between cost and outcomes for the three proposed methods of treating AOMd. This will be done for the short term (14 days) to assess the cost-effectiveness of immediate topically or delayed orally administered antibiotics compared with immediate orally administered antibiotics, and the longer term (3 months) in order to capture the effects of any recurrence of symptoms and lasting side effects. The perspectives will be from the NHS, parents/carers, and lost productivity due to time off work and school.

During the trial, participants will complete a symptom diary which will record any use of healthcare resources not available from the GP notes (for example, community care and the use of 111 and walk-in centres) and non-healthcare costs incurred by parents/carers during the first 14 days such as travel costs, purchase of over-the-counter medications, childcare and loss of earnings due to time off work. We will also ask about time off nursery/school.

### Qualitative data collection

The qualitative evaluation will explore the views and experiences of the trial processes, the acceptability of the different treatment options, and the barriers and facilitators to their use within, and future uptake outside the trial.

Purposive sampling [23] will select participants in order to capture maximum variation in views and experiences of a range of parents and primary care professionals. From parents who agree to trial participation and the interview, a purposive sample will be drawn in relation to site, arm of the trial, and socio-demographic variables such as socio-economic status. Parents who decline trial participation will also be invited to be interviewed. Primary care professionals involved in trial processes will also be purposively sampled in relation to site. Sample sizes will be determined by data saturation [23], such that no new themes are emerging from the data by the end of data collection. We anticipate including up to 20 clinicians, 20 participant parent interviews and 15 parent decliner/withdrawal telephone interviews.

In-depth interviews will be conducted with participating parents (from all arms of the trial) 14 days after randomisation [24]. Interviews with parents who declined to participate will be conducted within 7 of declining. These will be conducted by telephone at a time of the participant’s choosing. Interviews with primary care professionals will be conducted after 3–6 months of involvement in the trial to try to capture those with experience of recruitment. A flexible topic guide will be devised to ensure that the primary issues are covered across all interviews, but it will incorporate considerable flexibility to enable participants to introduce unanticipated issues, and they will be modified to reflect findings as they emerge. The researcher will use open-ended questioning techniques to elicit participants’ experiences and views of key events and participants will be asked to provide examples. Primary care professional’s interviews are expected to last around 30–45 min, parent interviews 30–40 min and those with parents who decline trial participation 10–20 min. Interviews will be recorded using a digital voice recorder, transcribed and anonymised to protect confidentiality.

### Trial oversight

The study is overseen by a Trial Management Group that meet on a monthly basis and consist of the chief investigator (CI), grant holders, study sponsor and any other staff responsible for the delivery of the trial. The Trial Steering Committee (TSC) provides independent supervision of the trial and oversees trial progress. The TSC consists of an independent chair and three other independent members including a clinical trialist a statistician, a Patient and Public Involvement (PPI) representative and the CI. The Data Monitoring Committee (DMC) monitors patient safety and trial data efficacy and consists of an independent chair, three other independent members and the CI.

All serious adverse events (SAE’S) are recorded and notified as appropriate to the relevant authorities.

### PPI

A comprehensive programme of PPI engagement was conducted during the set-up stage to inform the development of the symptom-diary data collection, of the parent- and child-facing trial documentation. PPI contributors attend both the Trial Management Group (TMG) and Trial Steering Committee (TSC) meetings, providing ongoing guidance. PPI members will help identify non-academic dissemination avenues, and will advise on materials for press releases, print media, social media and parent-facing materials, including presentation of results using a parent/child-friendly animation.

### Data management and confidentiality

Study data is collected and stored using the TRANSFoRm [19] GCP-validated clinical trial platform that is integrated into the GP’s EHR system. Once a patient presents to the GP with one of the specified otitis media disease codes, eligibility screening is run in the background on their EHR. Should the patient be found suitable for inclusion in the study, the GP is asked to consent the patient/legal guardian. No data is captured in TRANSFoRm until the parent/legal guardian has consented to theirs and their child’s participation. The data is captured through electronic Case Report Forms (eCRFs) completed through the EHR system, and the Patient Reported Outcome Measures (PROMs) completed at set time points by the patient through a web portal on their mobile devices, tablets or desktop computers at home. The data is transmitted using a secure connection and stored inside an encrypted database hosted at King’s College London. A subset of participant information is additionally stored in the REDCap database hosted at the University of Bristol, added by a member of the REST study team.

Both the TRANSFoRm electronic platform and REDCap incorporate data entry and validation rules to reduce data-entry errors and double data entry. Trial staff will ensure that participant anonymity is maintained through protective and secure handling and storage of patient information at the trial centre’s. Data will be anonymised as soon as it is practical to do so in line with the Data Protection Act 1998. Participants’ data is securely held on the databases in line with data protection legislation.

To comply with the Fifth Principle of the Data Protection Act 1998 (this process will be reviewed and updated accordingly with any updates to the guidelines), personal data will not be kept for longer than is required for the purpose for which it has been acquired. Data will be held in compliance with the sponsor’s standard operating procedures.

### Sample size

Our previous trial compared immediate with delayed antibiotic use [1]. Children with AOMd took a median of 3 days (interquartile range (IQR) 2, 4) to achieve the REST primary outcome. Our PPI advised a 1.25-day non-inferiority margin (equivalent to an absolute difference in cure rate of 19.5% at 3 days). A two-group non-inferiority trial normally assumes a 2.5% one-sided Type I error. Using a 1.25% Type I error to detect non-inferiority for two comparisons with 90% power, complete outcome data needed for 106 per arm is 399 with 20% attrition.

### Statistical analysis

A flow of participants through the trial will be summarised in a Consolidated Standards of Reporting Trials (CONSORT) diagram. Descriptive statistics of baseline clinical and socio-demographic characteristics will be presented to describe the study sample and to ascertain comparability of the randomisation groups.

Data from the internal pilot phase of the study will be assessed against predefined/pre-agreed stop/go criteria to inform the decision as to whether to continue the trial to the ‘main’ phase. The proposed ‘traffic light’ (stop/go) criteria are based on descriptive statistics summarising recruitment, retention and adherence.

The primary analysis will be carried out under the intention-to-treat (ITT) principle, analysing participants as randomised without the imputation of missing data. Kaplan-Meier survival curves will be plotted to depict the probability of symptom resolution over time. Symptom resolution over the 14 days of follow-up will be compared between children allocated to immediate oral antibiotics and those allocated to each of the other treatment groups using a Cox proportional hazards regression model, adjusted for age (stratification variable). The primary outcome will also be analysed using an Accelerated Failure Time (AFT) model, which has previously been recommended for studies of resolution of infectious diseases as previous research has suggested that symptoms of AOM will be resolved in 90% of children by day 8.

The proportion of participants in the immediate topically and delayed orally administered antibiotics arms who achieve symptom resolution within 3 days will be compared (separately) to those in the immediate orally administered antibiotics arm. The absolute difference will be calculated and reported alongside the associated confidence interval, it will then be reported as to whether or not the lower limit of the confidence interval lies within the maximum unimportant difference.

Analysis of secondary outcomes will utilise regression models appropriate for the nature of the outcome measure (i.e. logistic regression for binary outcomes, Poisson or negative binomial regression for count data).

The primary analysis model will be repeated but with the outcome of symptom resolution being defined as when all symptoms are rated as being ‘normal/none’, ‘very slight problem’ or ‘slight problem’ (compared to the primary outcome of symptom resolution being defined as all symptoms being rated as ‘normal/none’ or ‘very slight problem’). The primary analysis will also be repeated under the per-protocol approach (rather than ITT). The sensitivity of the primary analysis to the impact of missing data will also be explored by repeating the analysis after the imputation of missing primary outcome data. The primary analysis and AFT model will be repeated with additional adjustment for any prognostic variable showing a marked imbalance at baseline (ascertained using descriptive statistics). Baseline characteristics will be investigated as potential treatment effect moderators, this will be done by including treatment group by moderator interaction terms into the primary analysis model (individually).

### Economic data analysis

The primary economic evaluation will explore the relationship between cost and outcome for the three treatments for AOMd (immediate topically, delayed orally and immediately administered oral antibiotics) from an NHS perspective at 14 days post randomisation. This will take the form of a simple comparison of NHS costs and outcomes over a period of 2 weeks from randomisation.

A secondary cost analysis will evaluate the difference in NHS secondary care costs between the trial arms for the 3 months following randomisation.

All resources will be valued using unit costs from established sources. These will include Unit Costs of Health and Social Care [25] for primary and community care, NHS Reference Costs [26] for hospital care and the BNFC [27] for prescribed medication.

Differences in NHS resources and costs between the arms will be analysed initially using Ordinary Least Squares (OLS) regression. The distribution of residuals from the regression models will then be examined and a decision will be made as to whether OLS is appropriate or another type of regression model should be considered (e.g. Generalised Linear Models (GLM)).

A cost-consequence analysis will then be conducted in which the costs to the NHS of the three treatments at 14 days post randomisation will be compared with the primary clinical outcome. Areas of uncertainty in assumptions will be subjected to sensitivity analyses to test the robustness of the results.

### Qualitative data analysis

Interview transcripts will be imported into NVivo 12 qualitative data analysis software. Analysis will begin shortly after data collection starts and will be ongoing and iterative – informing further data collection and identifying changes needed to the topic guide. Thematic analysis [28], utilising a data-driven inductive approach, will be used to identify and analyse patterns and themes of particular salience for participants and across the dataset using constant-comparison techniques [29, 30]. A subset of transcripts will be independently double-coded by members of the team (CC and JH); any discrepancies will be discussed within the team and resolved to achieve coding consensus and maximal rigour.

### Blinding

Once the allocation is revealed, neither clinicians nor the child participant or their parent/legal guardian will remain blind to their allocated treatment. Codes will be assigned to the database, which will preserve blinding of study personnel. The senior statistician will remain blind to knowledge of which treatment is represented by each treatment code until the final results have been shared with the Data Monitoring Committee (DMC). Emergency unblinding will not be necessary in this trial since it is an open study.

### Dissemination

We will publish the trial results in peer-reviewed journals and present at national and international meetings. With the assistance of our collaborators and PPI we will disseminate the study findings to an international audience. All participants will be offered a lay summary of the main findings of the study.

## Discussion

This article outlines a pragmatic, three-arm, individually randomised trial, which aims to recruit from 175 GP practices across the UK. The aim is to establish evidence for the non-inferiority of: (1) immediate topical ear drops, ciprofloxacin 0.3%; (2) delayed orally administered amoxicillin (clarithromycin) or (3) immediate orally administered amoxicillin (clarithromycin). The non-inferiority will be based on time to resolution of all significant symptoms. Secondary outcomes will include cost-effectiveness, duration of moderately bad or worse symptoms and repeat AOMd episodes.

Recruitment of 399 children with AOMd over a 22-month period presented challenges around the design of the REST study. Only two to three patients’ children were expected to present at each GP practice over the 22-month recruitment period, requiring at least 175 GP practices to take part in the study.

In order to make this study feasible we needed to consider novel and streamline mechanism to facilitate greenlighting of sites, training of site staff, identification of eligible participants and the collection of high-quality data.

We employed several different strategies in order to address these elements of the study, these included:

- Development of a remote training platform to deliver REST study training to GPs and staff at recruiting practices
- A light-touch Green Light Process (GLP), maximising the number of staff at the GP practices able to recruit to the REST study by providing quick and accessible study-specific training

We used the TRANSFoRm electronic platform, (integrates into the health records) to automatically create a pop-up alert to the clinician when an eligible patient presents. The system automatically checked the participant’s eligibility and part-filled the study eCRFs reducing the efforts of the clinician to record study data.

### Trial status

Currently, 50 SystmOne GP practices have been greenlighted across seven CRN regions in the UK. The first child was recruited to the study on 5 April 2019 with recruitment currently ongoing. A total of 22 children were recruited to the REST study, with recruitment being closed on 31 May 2020.

Protocol: Version 7.0, 31 October 2018

Open to recruitment: 7 April 2019

Planned recruitment closure date: 31 March 2020

## Supplementary information

**Additional file 1.** REST Standard Protocol Items: Recommendations for Interventional Trials (SPIRIT) Checklist.

**Additional file 2.** The Runny Ear Study parent information sheet.

**Additional file 3.** REST study microbiology protocol.

**Additional file 4.** Agree-to-fund letter from the National Institute for Health Research (NIHR) Health Technology Assessment (NIHR HTA).

**Additional file 5.** Confirmation of favourable opinion by Ethics Commitee Letter.

## Data Availability

The datasets analysed during the current study will be available from the corresponding author on reasonable request.

## Abbreviations

AOMd: Acute otitis media with discharge
AE: Adverse event
CI: Chief investigator
CRF: Case Report Form
EHR: Electronic Health Record
GP: General practitioner
NHS: National Health Service
NICE: National Institute of Health and Social Care
PI: Principal investigator
TMG: Trial Management Group
TSC: Trial Steering Committee
DMC: Data Monitoring Committee
