# Supplementary material for: A multi-centre, pragmatic, three-arm, individually randomised, non-inferiority, open trial to compare immediate orally administered, immediate topically administered or delayed orally administered antibiotics for acute otitis media with discharge in children: The Runny Ear Study (REST): study protocol
Source: Trials. 2020 Jun 3;21:463. doi: 10.1186/s13063-020-04419-7 (PMC7268414; doi:10.1186/s13063-020-04419-7)
Supplement: Supplementary file 2 — Additional file 2. The Runny Ear Study parent information sheet. [file 13063_2020_4419_MOESM2_ESM.pdf]

the runny ear study

The Runny Ear STudy (REST)

FULL PARENT INFORMATION SHEET

Participation in the study is completely voluntary and if you do decide not to take part, this will not affect or change the treatment and care your child receives from the doctor or nurse. The study is for research purposes and your consent is needed before you and your child can take part.

WHAT IS THE PURPOSE OF THE STUDY?
Middle ear infections are common painful infections in children. Germs multiply in the middle ear causing a build-up of pressure that stretches the ear drum. In around 1 in 7 children, the ear drum bursts, releasing a liquid.

This study is looking at the best way to treat children with a middle ear infection, medically known as acute otitis media (AOM). At the moment nearly all UK children with AOM and discharge (AOMd) seen by their GP or nurse are treated with antibiotics by mouth. However, taking antibiotics by mouth can cause side-effects like rashes, diarrhoea and vomiting and more rarely, severe allergic reactions. They can also make the germs in a child's body resistant to antibiotics.

However, it may be possible to use alternative treatments for ear discharge:

- Using antibiotic eardrops: research has already shown that these are better than antibiotics by mouth for children with runny ears and grommets, probably because the antibiotics are given directly to the place they are most needed. The drops can be used for AOMd with a burst ear drum in the same way.
- Using a "just in case" or 'delayed' antibiotic prescription (where parents are advised to wait to see if the child's infection improves without antibiotics): our studies in other infections suggest this can be just as effective and safe, but with fewer side-effects.

The GP or nurse will randomly allocate your child to one of three treatments to treat AOMd. The treatment your child will receive is down to chance, like tossing a coin.

In this study we would like to find out whether giving an antibiotic ear drop or a delayed antibiotic by mouth is as good as immediately giving antibiotics by mouth for children with AOMd. AOMd is a common and painful infection for children so it is important that any new treatment works at least as well as the standard, immediate antibiotic treatment.

WHY HAVE MY CHILD AND I BEEN INVITED TO TAKE PART?
We are looking for 400 children age 12 months to 16 years to take part in this study. Your GP or nurse will have invited you and your child to take part in this study because your child has AOMd. Taking part in this study will not affect the usual care your child receives from their GP for any other conditions.

WHAT ARE THE ANTIBIOTIC EAR DROPS AND ARE THEY SAFE?
We are using ciprofloxacin eye drops (Ciloxan 0.3%) as the study antibiotic ear drops. Ciprofloxacin is widely available in the UK and is safe to be used in the ears as well eyes. Ciprofloxacin is the most commonly used treatment for external ear infections. Ear drops are as safe as other medicines commonly used to treat ear infections in children, such as antibiotics by mouth. We do not expect any children taking part in this trial to have any serious side-effects if they are allocated to receive the drops. A rash or itching of the skin in the outer ear may occur, but this will stop when the treatment is finished.

In rare cases your child may be allergic to ciprofloxacin. If your child is short of breath or is wheezing, or their face, lips or tongue start to swell, or they develop a rash, they need immediate medical treatment.

WHAT HAPPENS IF MY CHILD AND I TAKE PART?

- You will be asked some questions about your child's illness and general health.
- The doctor or nurse will look in your child's ears and check if it's safe for them to take part in the study.
- You will be asked to sign a consent form for you and your child to take part in the study (if your child aged 6-16 years can understand the study and what is involved for them they may sign their own consent form).
- Your child will be randomly allocated to one of the three treatment groups; immediate oral antibiotics, delayed oral antibiotics or ear drops.
- If your child is allocated to the ear drops group, you will be shown how to give them to your child until their ear discharge is better.
- You will be asked to complete a questionnaire (online or on paper) about your child's symptoms, ear discharge and any antibiotic or oral painkilling medicines, to be filled in today and for the next 13 days (14 days in total).
- The study team will 'phone you as soon as they get your details and up to a maximum of 4 more times over the next 13 days to answer any questions and collect information from the paper questionnaire. If you've chosen to complete the questionnaire online, we will call you to check if there are any problems and answer any questions you may have.
- If you consent to collect your child's stool sample, the study team will post you a sample collection kit and ask you to collect a stool sample on day 14 of the study. The study team will pre-label the stool sample swab with your child's study ID - you will be asked to write the date on the sample tube when it is taken. It is very important you write the exact date that you take the sample. The research nurse will remind you about the stool sample during the follow up calls. At 3 months another stool sample kit will be sent to you to collect another sample.
- After 3 months, you will either be sent a link to an online questionnaire or sent a paper copy of the questionnaire (with a pre-paid return envelope), for you to complete. The questionnaire is about your child's general health.
- With your permission NHS-approved researchers will look at your child's medical records after 3 months, to collect information on any other ear discharge episodes your child may have had (you do not need to be present).
- In appreciation of you and your child's time in taking part in the study, we will send you High Street shopping vouchers at the end of the study. Children aged 10-16 will receive a £10 voucher for taking part and those under 10 years will receive a piggy bank.
- You might be invited to be interviewed about your trial experiences at a later date (only some parents will be invited) and participation is optional.
- Most children with runny ears recover within one week but up to 30% can still be expected to have some pain, and up to 20% to still have some discharge. We suggest you contact your GP if your child's symptoms are not improving by day 7, or sooner if they are getting worse or you have any concerns.

WHAT HAPPENS IF I TAKE PART IN AN INTERVIEW?

- A researcher **might** phone to invite you to be interviewed for around 30 minutes (but no more than 45 minutes). They will answer your questions and if you agree, arrange a convenient time for the telephone interview.
- If you are interviewed, you will be asked about your views and experiences of the way your child's ear pain was treated by the doctor and how you think children's ear pain should be treated in the future. With your permission, the researcher **might** audio-record the interview.
- All the interviews conducted as part of this study will be audio-recorded and these digital audio recordings will be stored securely on encrypted and password protected drives separate from any personal information held about you. All the audio recordings will be transcribed, and during this process identifiable information will be removed
- Not all parents will be invited to take part, but if you do take part in one of these interviews you will receive a £10 voucher as a token of our thanks for your time.

WHAT ARE THE BENEFITS FOR ME AND MY CHILD?

The NHS has paid for and approved this study because evidence suggests the treatment might work. We cannot promise that your child will benefit directly, but you will be helping research to improve future treatment for children with ear discharge.

WHAT ARE THE RISKS FOR ME AND MY CHILD?

- As mentioned above, there are possible allergic reactions to the antibiotic drops or antibiotics taken by mouth, but the medicines are routinely prescribed in the UK and are considered safe to use for children.
- The advice to gently press on the outer ear flap to help the ear drops flow into the ear hole may cause discomfort to some children. If this happens, it is not necessary to continue using this “pumping” method but to simply keep your child’s head tilted to one side for a few extra minutes, to make sure the drops have reached the ear drum.
- The collection of the stool samples may prove to be a sensitive issue with your child or yourself, and therefore, the stool sample collection is optional. Your child’s participation in the study will not be affected if a sample cannot be obtained.

WHAT ELSE SHOULD I KNOW?

- Your child could receive antibiotic ear drops, delayed oral antibiotics or immediate oral antibiotics. We do not yet know whether using antibiotic eardrops is better than oral antibiotics or just in case antibiotics.
- You can still give your child painkillers. **Taking part in the study does not affect the care or treatment your child would normally receive from the doctor or nurse.**
- If your child is on other medication which stops them taking part in the study, the doctor or nurse will tell you.
- If you might be away on holiday or otherwise unavailable for us to reach you by telephone during the 14-day follow-up period, you might not be able to take part.
- If you have **any** problems completing the symptom questionnaire or giving the medicines, please do not worry that this will affect your child’s participation in the study. You can contact the study team if you have any questions at all – please see contact details listed on the following page.
- Stool samples will be sent to Southmead Hospital (North Bristol NHS Trust) in a Post Office-safe box designed for the transport of material. This will be placed inside a biohazard bag, which fits requirements of the UN3373 regulations on transport of biological substances.The stool sample collected from your child will be tested for antibiotic resistance in E.coli (a common bug or microbe which is found in stools) and is only used for research purposes. Your doctor will not routinely receive any results of this test. In the unlikely event that a microbe is found that could affect the health of your child the lab will contact your GP.
- After the stool samples have been tested they will be kept at the laboratory for the duration of the study. If you have consented to your child’s stool samples being used in future research related to microbes then the samples will be stored securely at the laboratory. If you have not consented to this, the samples will enter the normal disposal process.
- All participating GP practices will have their time compensated for participation in this study.

WHAT HAPPENS TO MY CHILD’S AND MY OWN INFORMATION?

When you and your child are recruited to the study, the GP will use an online, secure software system called TRANSFoRm, to extract information from your child’s medical records and enter information into the study research database. You and your child’s personal details which we use to contact you during the study follow-up period and to send out the 3-month stool sample kit and questionnaire, are transferred to a secure study administration database called REDCap at the University of Bristol. The information you give us will be securely stored for 15 years after the trial ends, at the University of Bristol and protected in accordance with the University’s guidelines and the Data Protection Act.

Personal details, e.g. names and addresses, will be stored separately to any other information you give us for this study and will be kept for a period of no more than 3 years after the trial ends. Only authorised and qualified staff will be able to look at the information. At the end of 3 years all of the identifiable information will be destroyed.

The findings of the study will be published in reports, scientific journals and presented at scientific conferences so that other people can learn from this research. Your information will be completely anonymous when this happens. Some quotes from the interviews may be published, but no names or other personal details will be used.

With your permission, data collected in this trial may be used in future ethically approved studies on the understanding that all shared information will be shared anonymously with other researchers. It will continue to be kept securely and remain confidential.

WHAT HAPPENS IF I DECIDE NOT TO TAKE PART OR DON'T WANT TO CARRY ON?
It's entirely your choice whether to take part. If you decide not to take part or you opt out after having joined the study, your child will still receive their usual medical care for ear infection.

You can stop taking part in the study **at any time** by telling the doctor or nurse or letting the research study team know in the following ways:-

- Phone: 0117 3314513 (there is an answerphone on this line)
- Text: 07787 666502
- Email: rest-trial@bristol.ac.uk

Although it would be useful to know why, you do not have to give us any reason. We would like to use any information collected up until that point, unless you tell us you do not want any of your information to be used.

We would like to interview a few people who decide not to take part about their reasons. If you are happy to do this, a researcher will arrange a convenient time for a short (about 20 minutes) telephone interview and we will send you a £10 High Street voucher as a thank you.

WHAT IF SOMETHING GOES WRONG?
In the unlikely event that something goes wrong please contact the study team and / or your local GP surgery. Bristol University has taken out an insurance policy and if something goes wrong and it is related to taking part in the study, you may be eligible for compensation. This does not affect your statutory legal rights.

WHO IS ORGANISING & FUNDING THE STUDY?
The study is organised by researchers at the Universities of Bristol, Southampton and Imperial College, London. The Research Directors are Professor Alastair Hay, University of Bristol (Alastair.Hay@bristol.ac.uk*)* and Professor Michael Moore, University of Southampton (mvm198@southampton.ac.uk).

This study has been funded by the National Institute for Health Research's Health Technology Assessment programme (# 16/85/01).

WHO HAS REVIEWED THE STUDY?
All research in the NHS is looked at by an independent group of people, called a Research Ethics Committee, to protect your interests. This study has been reviewed and given a favourable opinion by the South Central - Oxford B Research Ethics Committee.

CONTACT FOR FURTHER INFORMATION
If you require more information or wish to discuss the study further, please contact Kathryn Curtis, Study Manager **(0117 3313906 / email: Kathryn.Curtis@bristol.ac.uk)** or Sue Harris, Study Research Nurse (**0117 3314513 (there is an answerphone on this line) / email: Sue.D.Harris@bristol.ac.uk)**.

WHAT IF I WANT TO MAKE A COMPLAINT?
The Patient Advice and Liaison Service (PALS) can provide general advice about taking part in research. They can provide help and support with a range of queries. PALS can be contacted on 0117 9003433 or pals@bristolpct.nhs.uk.

If you wish to make a formal complaint please contact: Kat Tucker, Complaints and Freedom of Information Manager, NHS Bristol, South Plaza, Marlborough Street, Bristol, BS1 3NX or telephone: 0117 900 2494.

THANK YOU FOR READING THIS.

IF YOU WOULD LIKE TO TAKE PART OR WOULD LIKE MORE INFORMATION OR ADVICE PLEASE TALK TO THE RECEPTIONIST, DOCTOR OR NURSE.

University of BRISTOL | UNIVERSITY OF Southampton | Bristol Randomised Trials Collaboration | REST the runny ear study | Funded by NHS National Institute for Health Research

IRAS ID: 229293

The Runny Ear Study is funded by the National Institute for Health Research's HTA Programme HTA 16/85/01
