# Supplementary material for: A multi-centre, pragmatic, three-arm, individually randomised, non-inferiority, open trial to compare immediate orally administered, immediate topically administered or delayed orally administered antibiotics for acute otitis media with discharge in children: The Runny Ear Study (REST): study protocol
Source: Trials. 2020 Jun 3;21:463. doi: 10.1186/s13063-020-04419-7 (PMC7268414; doi:10.1186/s13063-020-04419-7)
Supplement: Supplementary file 3 — Additional file 3. REST study microbiology protocol. [file 13063_2020_4419_MOESM3_ESM.pdf]

REST study microbiology protocol

Background

The REST study is a HTA-funded project to investigate the clinical effectiveness and economic impact of immediate topical or delayed oral antibiotics compared with immediate oral antibiotics for symptom duration in children presenting to primary care with acute otitis media with discharge AOMd). The primary objective is to determine whether either ciprofloxacin 0.3% drops, or delayed oral amoxicillin (clarithromycin if penicillin allergic or other suitable oral antibiotic as chosen by the GP), is non-inferior to current usual care (immediate oral antibiotics) for overall illness duration in children with AOMd presenting to primary care. As part of the study, faeces samples will be submitted to assess whether there is any evidence of differences in susceptibility of *Escherichia coli* to study antibiotics after treatment. Patient samples will be collected by subjects and/or their carers approximately 14 days and 3 months after treatment and submitted to the Antimicrobial Reference Laboratory, Infection Sciences, Southmead Hospital for analysis.

Specimen receipt

Swab samples will be received by Royal Mail direct from study subjects. Swabs in a tube containing transport gel will be packed in a secondary container with absorbent material (SpeciSafe, Alpha Labs SH060055) packed in a UN 3373 'MailTuff' envelope. Both the outer envelope and the SpeciSafe container will be labelled with the participant ID number, the sample (day 14 or month 3) will be specified on the SpeciSafe container. The date that the sample was taken should be written on the tube containing the swab.

Wear nitrile or latex gloves when unpacking the specimen. Label the swab tube with the participant ID number as soon as soon as it is removed from the SpeciSafe container.

Swab processing

Label a Microbank cryovial (www.pro-lab.co.uk/products/microbanktm) with the Study ID number and the collection date (written on the swab sleeve) or 3 month/day14 as appropriate (on SpeciSafe container) if date is missing. Remove the swab from its sleeve and disperse the sample from the swab tip into the glycerol broth in the cryovial by rotating the swab gently in the beads at the bottom of the Microbank tube for approximately 15 seconds. Remove the swab and inoculate onto a BD CHROMagar Orientation Medium (www.bd.com/resource.aspx?IDX=9020) chromogenic plate labelled with Study ID number using standard streaking bacteriological technique. Incubate the plate aerobically at 37°C for 18-24 hours. Cap and store the cryovial at -80°C.

Reading CHROMagar plate and susceptibility testing

After incubation, examine CHROMagar plates for colonies with morphology typical of *E. coli* on this agar (medium to large-sized, transparent, dark rose to pink). Select up to six morphologically distinct

colonies and streak for purity on to a CLED plate. Confirm identity by MALDI-TOF spectroscopy (Bruker Biotyper). Determine susceptibility of *E. coli* cultures to ampicillin, ciprofloxacin and erythromycin (as surrogate for clarithromycin) by disk diffusion using EUCAST methodology.

Store tested isolates in Microbank cryovials labelled with Study ID number and the collection date (as above) and Ec1, Ec2, Ec3 etc.

Recording results

Record study ID number, Day 14 or 3 month sample, date sample collected, date sample received and date sample processed. For each *E. coli* isolate, MALDI-TOF score, ampicillin ciprofloxacin and erythromycin zone diameter and deduced susceptibility on the REST study microbiology data collection form. Record location of cryovials in REST freezer boxes to aid location and retrieval. Transfer results to Excel spreadsheet at the earliest opportunity.

Antimicrobial Reference Laboratory, Southmead Hospital, Bristol

REST study microbiology data collection form

Sample details

Study ID number …………………..

Sample is - Day 14 / Month 3 (circle as appropriate)

Date sample collected ……………………..

Freezer box location (sample cryovial) ………………………

Isolate details

Isolate Ec1:

MALDI-TOF score …………………

Ampicillin zone diameter ……………………… = S R (circle as appropriate)

Ciprofloxacin zone diameter ……………………. = S I R (circle as appropriate)

Erythromycin zone diameter, …………………

Freezer box location (isolate cryovial) ………………………

Isolate Ec2:

MALDI-TOF score …………………

Ampicillin zone diameter ……………………… = S R (circle as appropriate)

Ciprofloxacin zone diameter ……………………. = S I R (circle as appropriate)

Erythromycin zone diameter, …………………

Freezer box location (isolate cryovial) ………………………

PTO

Isolate Ec3:

MALDI-TOF score …………………

Ampicillin zone diameter ……………………… = S R (circle as appropriate)

Ciprofloxacin zone diameter ……………………. = S I R (circle as appropriate)

Erythromycin zone diameter, …………………

Freezer box location (isolate cryovial) ………………………

Isolate Ec4:

MALDI-TOF score …………………

Ampicillin zone diameter ……………………… = S R (circle as appropriate)

Ciprofloxacin zone diameter ……………………. = S I R (circle as appropriate)

Erythromycin zone diameter, …………………

Freezer box location (isolate cryovial) ………………………

Isolate Ec5:

MALDI-TOF score …………………

Ampicillin zone diameter ……………………… = S R (circle as appropriate)

Ciprofloxacin zone diameter ……………………. = S I R (circle as appropriate)

Erythromycin zone diameter, …………………

Freezer box location (isolate cryovial) ………………………

Isolate Ec6:

MALDI-TOF score …………………

Ampicillin zone diameter ……………………… = S R (circle as appropriate)

Ciprofloxacin zone diameter ……………………. = S I R (circle as appropriate)

Erythromycin zone diameter, …………………

Freezer box location (isolate cryovial) ………………………
