# Supplementary material for: A multi-centre, pragmatic, three-arm, individually randomised, non-inferiority, open trial to compare immediate orally administered, immediate topically administered or delayed orally administered antibiotics for acute otitis media with discharge in children: The Runny Ear Study (REST): study protocol
Source: Trials. 2020 Jun 3;21:463. doi: 10.1186/s13063-020-04419-7 (PMC7268414; doi:10.1186/s13063-020-04419-7)
Supplement: Supplementary file 4 — Additional file 4. Agree-to-fund letter from the National Institute for Health Research (NIHR) Health Technology Assessment (NIHR HTA). [file 13063_2020_4419_MOESM4_ESM.pdf]

National Institute for Health Research

Evaluation, Trials and Studies Coordinating Centre
University of Southampton
Alpha House
Enterprise Road
Southampton
SO16 7NS

Tel: 023 8059 5586
Fax: 023 8059 5639
Email: info@netscc.ac.uk
Web: www.nets.nihr.ac.uk

Professor Alastair Hay
Professor of Primary Care
Centre for Academic Primary Care
Canynge Hall
39 Whatley Road
Bristol
Somerset
BS8 2PS

Direct Line: 02380 59 7462
Email: Netspostawardsetup@nihr.ac.uk
2 June 2017

Dear Professor Hay

HTA Project: 16/85/01 - Immediate oral, immediate topical or delayed oral antibiotics for acute otitis media with discharge (the Runny Ear STudy: REST)

Thank you for responding to the concerns raised by the Board. Following consideration of your revised application dated 18 May 2017, I am pleased to inform you that your above-titled proposal has been recommended for funding. Please would you inform your co-applicants of the decision.

<u>Revised Application Signatures</u>

We are unable to raise a contract without receiving an updated set of electronic signatures from those in key supporting roles (e.g. Head of Department, Finance) confirming approval of your revised application form and project description.

Please log into the MIS to complete the 'Provide Final App Signatures' task. Once all signatories have completed their individual electronic signature tasks they will show as 'Complete'. Submit the task by going to the 'Review and Submit' section of the task and pressing the 'Submit' button at the bottom of the page. Please aim to submit the task with all the required signatures within the next two weeks so that we may proceed to the contracting stage.

If any of the signatories need to be updated, e.g. due to staff changes, please inform me as soon as possible.

Clean Copy of Detailed Project Description

Please email me a copy of your detailed project description with all tracked changes accepted/removed, for inclusion in the contract.

Start-Up Information

I will be contacting you again soon to request key project information and to facilitate the timely start-up of your study.

Publicising Your Project

Please note that this award is not secure until the work has been contracted. Therefore, you must not publicise this award until you receive a fully executed contract from us.

Also, please note we are in Purdah due to the general election. This means that for the time being it is important there is no publicity of your project unless it is specifically for the purposes of participant recruitment. We anticipate Purdah to be in place until approximately 6 weeks after the general election.

Congratulations, and we wish you all success with your project.

If you have any queries please contact me on netspostawardsetup@nihr.ac.uk or 02380 59 7462.

Yours sincerely,

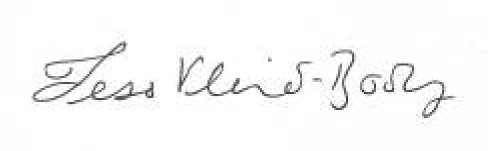

Miss Jess Vlieland-Boddy
The HTA Programme
