# Supplementary material for: A multi-centre, pragmatic, three-arm, individually randomised, non-inferiority, open trial to compare immediate orally administered, immediate topically administered or delayed orally administered antibiotics for acute otitis media with discharge in children: The Runny Ear Study (REST): study protocol
Source: Trials. 2020 Jun 3;21:463. doi: 10.1186/s13063-020-04419-7 (PMC7268414; doi:10.1186/s13063-020-04419-7)
Supplement: Supplementary file 5 — Additional file 5. Confirmation of favourable opinion by Ethics Commitee Letter. [file 13063_2020_4419_MOESM5_ESM.pdf]

Health Research Authority

South Central - Oxford B Research Ethics Committee

Whitefriars
Level 3, Block B
Lewin's Mead
Bristol
BS1 2NT

Telephone: 02071048058

> **Please note: This is the favourable opinion of the REC only and does not allow you to start your study at NHS sites in England until you receive HRA Approval**

22 May 2018

Dr Alastair Hay
University of Bristol
Canynge Hall, 39 Whatley Road
Bristol
BS8 2PS

Dear Dr Hay

**Study title:**	**Immediate oral, immediate topical or delayed oral antibiotics for acute otitis media with discharge (the Runny Ear STudy: REST)**
**REC reference:**	**18/SC/0181**
**Protocol number:**	**2814**
**EudraCT number:**	**2017-003635-10**
**IRAS project ID:**	**229293**

Thank you for your letter of 10th May 2018, responding to the Committee's request for further information on the above research and submitting revised documentation.

The further information has been considered on behalf of the Committee by the Chair, together with Dr Kim Cheetham.

We plan to publish your research summary wording for the above study on the HRA website, together with your contact details. Publication will be no earlier than three months from the date of this opinion letter. Should you wish to provide a substitute contact point, require further information, or wish to make a request to postpone publication, please contact hra.studyregistration@nhs.net outlining the reasons for your request.

Confirmation of ethical opinion

On behalf of the Committee, I am pleased to confirm a favourable ethical opinion for the above research on the basis described in the application form, protocol and supporting documentation as revised, subject to the conditions specified below.

- The Committee would like to offer some advice to the applicant:
  Applicant might want to consider whether the protocol should contain a list of circumstances in which the child should be withdrawn from the allocated treatment, and instead treated as the GP thinks best. That is, of course, the responsibility of any doctor whose patient is in a trial, but it might help to codify circumstances in which the trial treatment should be abandoned.

Conditions of the favourable opinion

The REC favourable opinion is subject to the following conditions being met prior to the start of the study.

Management permission must be obtained from each host organisation prior to the start of the study at the site concerned.

Management permission should be sought from all NHS organisations involved in the study in accordance with NHS research governance arrangements. Each NHS organisation must confirm through the signing of agreements and/or other documents that it has given permission for the research to proceed (except where explicitly specified otherwise).

Guidance on applying for HRA and HCRW Approval (England and Wales)/ NHS permission for research is available in the Integrated Research Application System, at www.hra.nhs.uk or at http://www.rdforum.nhs.uk.

Where a NHS organisation's role in the study is limited to identifying and referring potential participants to research sites ("participant identification centre"), guidance should be sought from the R&D office on the information it requires to give permission for this activity.

For non-NHS sites, site management permission should be obtained in accordance with the procedures of the relevant host organisation.

Sponsors are not required to notify the Committee of management permissions from host organisations

Registration of Clinical Trials

All clinical trials (defined as the first four categories on the IRAS filter page) must be registered on a publically accessible database within 6 weeks of recruitment of the first participant (for medical device studies, within the timeline determined by the current registration and publication trees).

There is no requirement to separately notify the REC but you should do so at the earliest opportunity e.g. when submitting an amendment. We will audit the registration details as part of the annual progress reporting process.

To ensure transparency in research, we strongly recommend that all research is registered but for non-clinical trials this is not currently mandatory.

If a sponsor wishes to request a deferral for study registration within the required timeframe, they should contact hra.studyregistration@nhs.net. The expectation is that all clinical trials will be registered, however, in exceptional circumstances non registration may be permissible with prior agreement from the HRA. Guidance on where to register is provided on the HRA website.

Clinical trial authorisation must be obtained from the Medicines and Healthcare products Regulatory Agency (MHRA).

It is the responsibility of the sponsor to ensure that all the conditions are complied with before the start of the study or its initiation at a particular site (as applicable).

Ethical review of research sites

NHS sites

The favourable opinion applies to all NHS sites listed in the application, subject to management permission being obtained from the NHS/HSC R&D office prior to the start of the study (see "Conditions of the favourable opinion" below).

Non-NHS sites

The Committee has not yet completed any site-specific assessment (SSA) for the non-NHS research site(s) taking part in this study. The favourable opinion does not therefore apply to any non-NHS site at present. We will write to you again as soon as an SSA application(s) has been reviewed. In the meantime no study procedures should be initiated at non-NHS sites.

Approved documents

The final list of documents reviewed and approved by the Committee is as follows:

Document	*Version*	*Date*
Covering letter on headed paper [Cover Letter]	v1.0	06 March 2018
Covering letter on headed paper [Cover Letter]	v2.0	10 May 2018
Details of any Data Monitoring Committee [DMSC Members]		01 December 2017
Evidence of Sponsor insurance or indemnity (non NHS Sponsors only) [Confirmation of Sponsor Insurance and indemnity]		26 January 2018
GP/consultant information sheets or letters [REST GP Information Sheet]	v0.3	06 March 2018
IRAS Application Form [IRAS_Form_09032018]		09 March 2018
IRAS Checklist XML [Checklist_14052018]		14 May 2018
Letter from statistician [Letter from statistician]		24 January 2018
Non-validated questionnaire [Symptom Questionnaire]	v1.0	06 March 2018
Other [SmPC Amoxicillin 250mg]		18 September 2017
Other [SmPC Amoxicillin 500mg]		18 September 2017

Other [SmPC Ciloxan 0.3%]		18 September 2017
Other [SmPC Clarithromycin 125mg]		18 September 2017
Other [SmPC Clarithromycin 250mg]		18 September 2017
Other [SmPC Clarithromycin 250mg Film coated tablets]		18 September 2017
Other [Invitation to parent interview- Qualitative Study]	v1.0	27 November 2017
Other [Invitation to clinician interview-Qualitative study]	v1.0	27 November 2017
Other [Clinician interview topic guide-Qualitative Study]	v1.0	27 November 2017
Other [Trial Parent interview topic guide- Qualitative Study]	v1.0	27 November 2017
Other [Decline parent interview topic guide- Qualitative Study]	v1.0	27 November 2017
Other [Health care practitioners interview information sheet-Qualitative Study]	v1.0	22 January 2018
Other [Decline parent interview information sheet-Qualitative Study]	v1.0	24 January 2018
Other [CV Professor Michael Moore]		02 December 2015
Other [Parent advice for ciprofloxacin]	v2.0	10 May 2018
Other [Parent advice for delayed antibiotics]	v2.0	10 May 2018
Other [Parents advice for immediate antibiotics]	v2.0	10 May 2018
Other [REST Stool Sample Instructions]	v2.0	10 May 2018
Other [Stool sample collection instructions-TRACKED CHANGED VERSION]	v2.0	10 May 2018
Other [Parent advice for ciprofloxacin-TRACKED CHANGED VERSION]	v2.0	10 May 2018
Other [Parent advice for ciprofloxacin-IN NEW MFC FORMAT]	v2.0	10 May 2018
Other [Parent advice for delayed antibiotics-TRACKED CHANGED VERSION]	v2.0	10 May 2018
Other [Parent advice for delayed antibiotics-IN NEW MFC FORMAT]	v2.0	10 May 2018
Other [Parents advice for immediate antibiotics-TRACKED CHANGED VERSION]	v2.0	10 May 2018
Other [Parents advice for immediate antibiotics-IN NEW MFC FORMAT]	v2.0	10 May 2018
Other [Protocol-TRACKED CHANGED VERSION]	v5.0	10 May 2018
Other [Appendix 1]	v1.0	10 May 2018
Other [REST mascot to accompany Patient Documents]	N/A	10 May 2018
Participant consent form [Telephone consent form]	v1.0	22 February 2018
Participant consent form [Child Assent form]	v1.0	22 February 2018
Participant consent form [Consent form]	v2.0	10 May 2018
Participant consent form [Consent form-TRACKED CHANGED VERSION]	v2.0	10 May 2018
Participant information sheet (PIS) [Parent Information Sheet]	v2.0	10 May 2018
Participant information sheet (PIS) [Parent information sheet-TRACKED CHANGED VERSION]	v2.0	10 May 2018
Participant information sheet (PIS) [Parent Summary Information Sheet]	v2.0	10 May 2018
Participant information sheet (PIS) [Parent Summary Information Sheet- TRACKED CHANGED VERSION]	v2.0	10 May 2018

Participant information sheet (PIS) [Child information sheet age 6-10]	v2.0	10 May 2018
Participant information sheet (PIS) [Child information sheet age 6-10-TRACKED CHANGED VERSION]	v2.0	10 May 2018
Participant information sheet (PIS) [Child information sheet age 11-16]	v2.0	10 May 2018
Participant information sheet (PIS) [Child information sheet age 11-16 -TRACKED CHANGED VERSION]	v2.0	10 May 2018
Referee's report or other scientific critique report [Referee Report]		09 March 2017
Research protocol or project proposal [Protocol]	v5.0	10 May 2018
Summary CV for Chief Investigator (CI) [Summary CV]		16 April 2015
Summary of product characteristics (SmPC) [Amoxicillin 125 mg]		24 January 2018
Summary, synopsis or diagram (flowchart) of protocol in non technical language [Trial workflow]	v2.0	28 February 2018
Validated questionnaire [OM-6 questionnaire]		

Statement of compliance

The Committee is constituted in accordance with the Governance Arrangements for Research Ethics Committees and complies fully with the Standard Operating Procedures for Research Ethics Committees in the UK.

After ethical review

Reporting requirements

The attached document *"After ethical review – guidance for researchers"* gives detailed guidance on reporting requirements for studies with a favourable opinion, including:

- Notifying substantial amendments
- Adding new sites and investigators
- Notification of serious breaches of the protocol
- Progress and safety reports
- Notifying the end of the study

The HRA website also provides guidance on these topics, which is updated in the light of changes in reporting requirements or procedures.

User Feedback

The Health Research Authority is continually striving to provide a high quality service to all applicants and sponsors. You are invited to give your view of the service you have received and the application procedure. If you wish to make your views known please use the feedback form available on the HRA website:
http://www.hra.nhs.uk/about-the-hra/governance/quality-assurance/

HRA Training

We are pleased to welcome researchers and R&D staff at our training days – see details at http://www.hra.nhs.uk/hra-training/

**18/SC/0181**	**Please quote this number on all correspondence**

With the Committee's best wishes for the success of this project.

Yours sincerely

pp V. Cornfield-Butler

Mr Chris Foy
Chair

Email:nrescommittee.southcentral-oxfordb@nhs.net

Enclosures: "After ethical review – guidance for researchers" *[SL-AR1]*

Copy to: *Dr Birgit Whitman*
Mrs Rachel Avery, Avon Primary Care Research Collaborative
